# Supplementary material for: Light-driven modulation of proximity-enhanced functionalities in hybrid nano-scale systems
Source: Nat Commun. 2025 Aug 7;16:7297. doi: 10.1038/s41467-025-62571-7 (PMC12332133; doi:10.1038/s41467-025-62571-7)
Supplement: Supplementary file 1 — Supplementary Information [file 41467_2025_62571_MOESM1_ESM.pdf]

Supporting information (SI) for
"Light-driven modulation of proximity-enhanced functionalities in hybrid nano-scale systems"

Mattia Benini[a,b,*], Umut Parlak[b], Sophie Bork[b], Jaka Strohsack[c], Richard Leven[b], David Gutnikov[b], Fabian Mertens[b], Evgeny Zhukov[b], Rajib Kumar Rakshit[a], Ilaria Bergenti[a], Andrea Droghetti[d], Andrei Shumilin[c,e], Tomaz Mertelj[c], Valentin Alek Dediu[a], Mirko Cinchetti[b,*]

a ISMN-CNR, Via Piero Gobetti 101, 40129 Bologna, Italy
b TU Dortmund University, Otto-Hahn-Straße 4, 44227 Dortmund, Germany
c Jozef Stefan Institute, Jamova Cesta 39, 1000 Ljubljana, Slovenia
d Universitá Ca' Foscari Venezia, Via Torino 155, 30170 Venezia Mestre, Italy
e Instituto de Ciencia Molecular (ICMol), Universitat de Valencia, c/Catedrático José Beltrán, 2, Paterna 46980, Spain

S.I Calculation of the fluence adsorbed by Cobalt and error estimations.

For each pump wavelength, λ, the deposited energy density in the cobalt $w_{Co}(\lambda)$ layer is calculated based on the static RT transmittance measurements of Co/C_{60} and Co/Al samples, reported in **Fig. S1 a**. These transmittance measurements were performed at RT with a custom-made setup. We employed a supercontinuum white-light source emitting in the range 360-2600 nm (its spectrum is given in **Fig. S1 b**) and acquired the transmitted light beam with a spectrometer detecting wavelengths in the range (360-1000nm). The apparent ≈0.95 transmittance of both samples in the range 360-380 is an artifact due to a lack of emitted power from the source, resulting in a very weak photon count in the detector.

To calculate $w_{Co}(\lambda)$ we assume that all the light not transmitted by the Co/Al sample is absorbed by the system. Moreover, we approximate the Co/Al transmittance (T) as a constant value of 0.95, meaning that 5% of the light is absorbed, independent of wavelength. Furthermore, we assume that the Al layer is fully oxidized by air exposure [1,2]. Thus, for the Co/Al sample, the deposited energy density is:

$$w_{Co} = \left(1 - T_{Co/Al}\right)F \approx 0.05F. \ (S1)$$

This quantity can be interpreted as the energy absorbed by the Co layer. In the case of the Co/C_{60} sample, we also account for the absorption by the C_{60} layer, which reduces the optical intensity reaching the Co layer. As the percentage of energy density absorbed by the Co layer (0.05) is known from the reference sample, we can estimate the amount of light transmitted by the C_{60} layer as:

$$T_{C60} = T_{Co/C60} + 0.05\ (S3) = \begin{cases} 0.75 + 0.05 = 0.8\ (\lambda = 450\ nm) \\ 0.80 + 0.05 = 0.85\ (\lambda = 400\ nm) \end{cases}\ (S2)$$

where $T_{Co/C60}$ is the measured transmittance of the Co/C_{60} sample (reported in **Fig. S1**). At 650 nm we assume negligible absorption by the C_{60} layer, as apparent from the transmittance measurement. Thus, the absorbed energy density of the Co layer is

$$w_{Co} = (1 - T_{C60})0.05F\ (S3)$$

The external pump fluence is given by

$$F[mJ/cm^2] = \frac{P[mW])}{rep.rate[Hz]\pi\left(\frac{d[cm^2]}{2}\right)^2}, (S4)$$

where P is the average pump power and d the beam spot diameter (at the focal position). The spot diameter at the sample position was measured by a beam profiler and estimated as the FWHM of the beam Gaussian profile. The diameter error estimation is about 3µm, accounting for an 8% relative error for the smallest diameter measured (36 µm for the 650 nm pump wavelength). The relative error of the power measurement is estimated to be 2% of the measured value. Hence, most of the error comes from the diameter estimation. Error analysis estimation gives for the smallest diameter measured, about 16% of the calculated fluence value. We then calculated the absolute error using this worst-case estimation.

The values of ν extrapolated by the damped sinusoidal fit of the TR MOKE signals of Co/C_{60} have an associated absolute error that is typically of the order 0.1 GHz, as extrapolated from the fit. We believe that such error is too low and does not take into account the effect of background removal or of the uncertainty in the absorbed fluence. Hence, we deliberately chose a fixed error value of 1 GHz. For the same reason we decided to apply a roughly 10 times increase in the estimated absolute error associated to the ν values extrapolated from the Co/Al data.

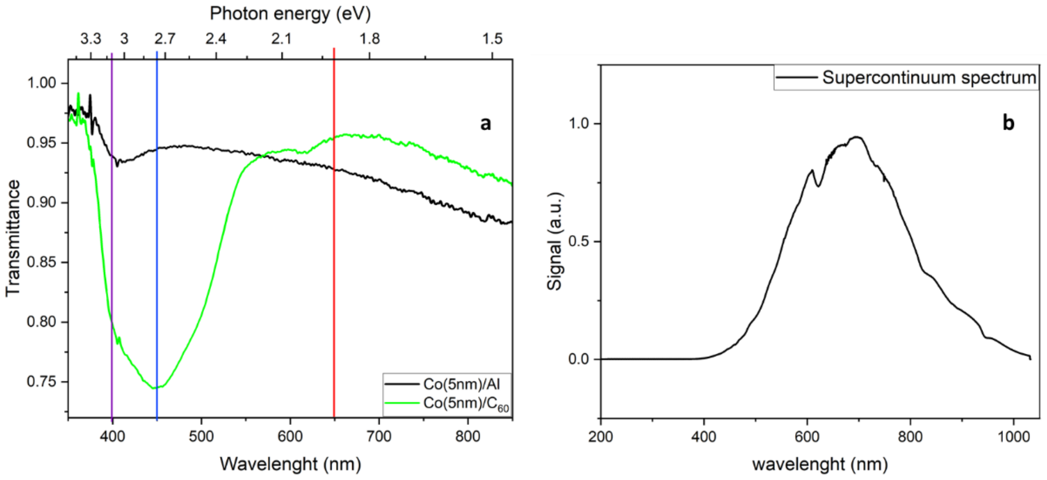

Figure S1 a, RT Transmittance of the Co/C_{60} and the reference Co/Al samples. The vertical lines indicate the selected pump pulse wavelengths used in the tr-MOKE measurements. **b**, Spectrum of the supercontinuum source used for the measurements.

S.II Time-resolved MOKE data

In this section, we report the raw data obtained in the tr-MOKE measurements. All the data were taken at a fixed temperature T = 80 K and with an external out-of-plane field of 0.5 T. All the tr-MOKE traces were fitted with the following function:

$$\theta_K(t) = \theta_0 + A_1 \exp\left(-\frac{t}{\tau_1}\right) + A_2 \exp\left(-\frac{t}{\tau_2}\right) + A_0 \exp\left(-\frac{t}{\tau_D}\right) \sin\big(2\pi\nu(t - t_0)\big) \quad (S5)$$

where the first two terms represent a background contribution, while the damped sinusoidal term corresponds to the magnetic oscillations that we want to extract from the measurements. The quantity $\theta_K(t)$ is actually $\Delta\theta_K(t) \propto \theta_K(t)_{Pump\ OFF} - \theta_K(t)_{Pump\ ON} = \theta_K(t)_{Pump\ ON}$ as the signal into the photodetector was balanced to 0 before the measurements[3]. All the data are labelled with respect to the pump fluence absorbed by the Co layer, $w_{Co}(\lambda_{pump})$. In **Fig. S2** we present the tr-MOKE data for Co/Al for pump wavelengths of 650 nm (1.9 eV) and 450 nm (2.75 eV). The tr-MOKE data of Co/C_{60} for pump wavelengths of 650 nm (1.9 eV), 450 nm (2.75 eV) and 400 nm (3.1 eV) are reported in **Fig. S3**. The extracted values of the oscillation amplitude A_0 and decay (damping) time τ_D for each sample at different pump wavelengths are reported in **Fig. S4**, along with the extracted values of

$\theta_K(t = t_0)$ where t_0 represents the time at which the pump and probe pulses temporally overlap.

First, we used the value of Kerr rotation at t_0 to confirm a linear dependence on w_{Co} (**Fig. S4 a**). The parameters A_0 and τ_D also exhibit a linear trend (see **Fig. S4 b-c**), as expected in the low-fluence regime. In general, Co interfaced with C_{60} systematically shows shorter τ_{D}s values compared to the reference Co/Al sample. We attribute this to an increased disorder of the magnetic configuration of the Co/C_{60} sample, which promotes faster decoherence of the local magnetization vector. This behaviour is predicted by recent theoretical studies on random anisotropy fields[4], which are present in the Co/C_{60} system[5].

For completeness, we also report in **Fig. S4 d** the tr-MOKE traces showing the ultrafast magnetization dynamics of the Co/C_{60} samples within a 10 ps window after optical excitation, recorded with a temporal resolution of 0.1 ps. The onset of oscillations following optical excitation is inherently linked to ultrafast magnetization dynamics, which involve multiple competing processes in the first few picoseconds. After sub-picosecond demagnetization, excess energy is first dissipated through electron-phonon relaxation on a few-picosecond timescale before significant heat transfer to the substrate occurs[6] . As a result, the early-stage magnetization response is highly complex and cannot be simply described as the sum of independent components.

However, our data confirm that oscillations become clearly visible only after ultrafast demagnetization has occurred, electron-phonon thermalization has largely taken place, and the magnetization begins to recover. This recovery process occurs on a timescale of tens of picoseconds, consistent with expectations from previous studies on ultrafast magnetization dynamics. The observed delay in the emergence of oscillations reflects the time required for the effective magnetic field to realign and stabilize.

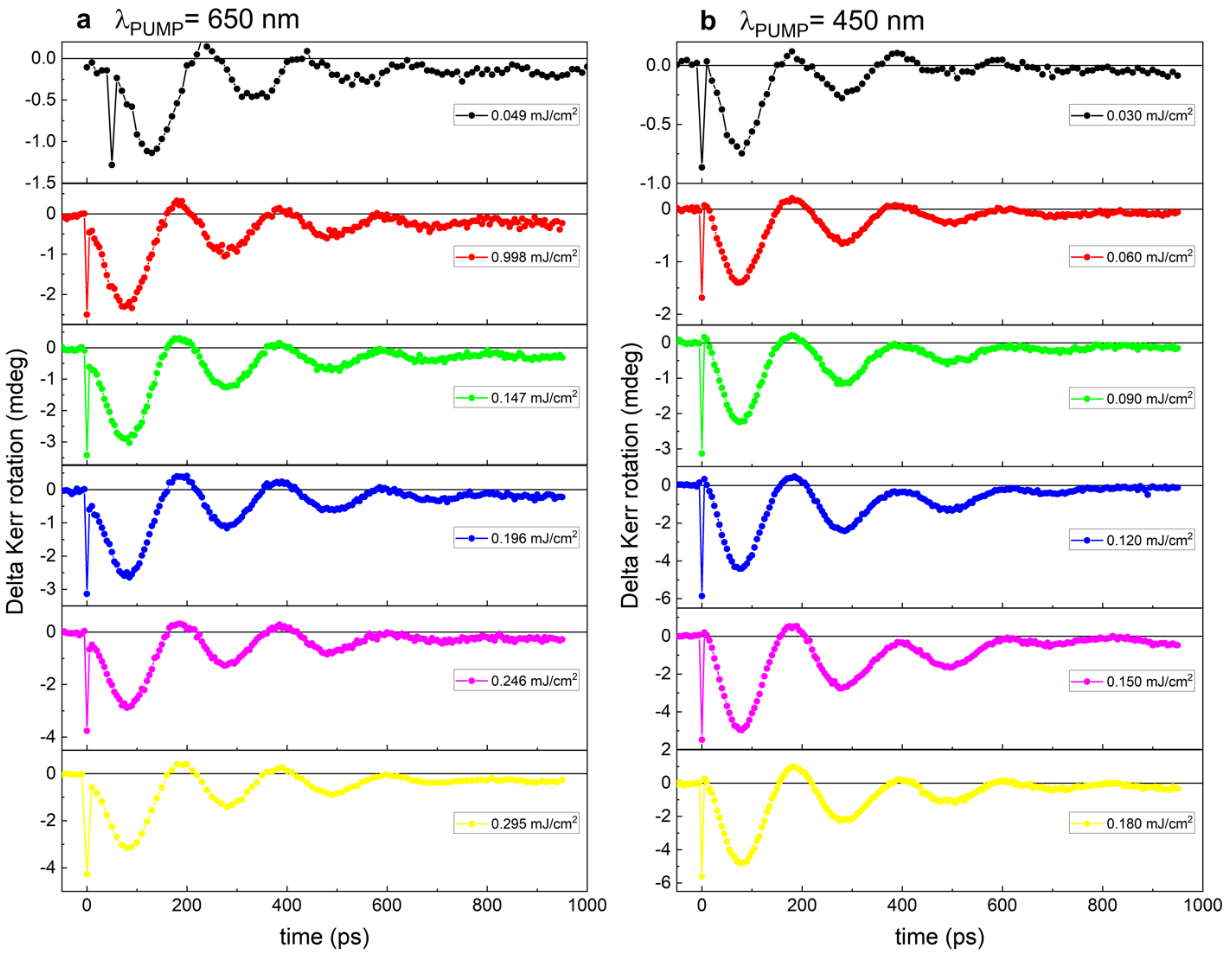

Figure S2 Raw tr-MOKE data obtained from the Co/Al sample at T=80K for pump wavelengths of **a**, 650 nm (1.9 eV), and **b**, 450 nm (2.75 eV).

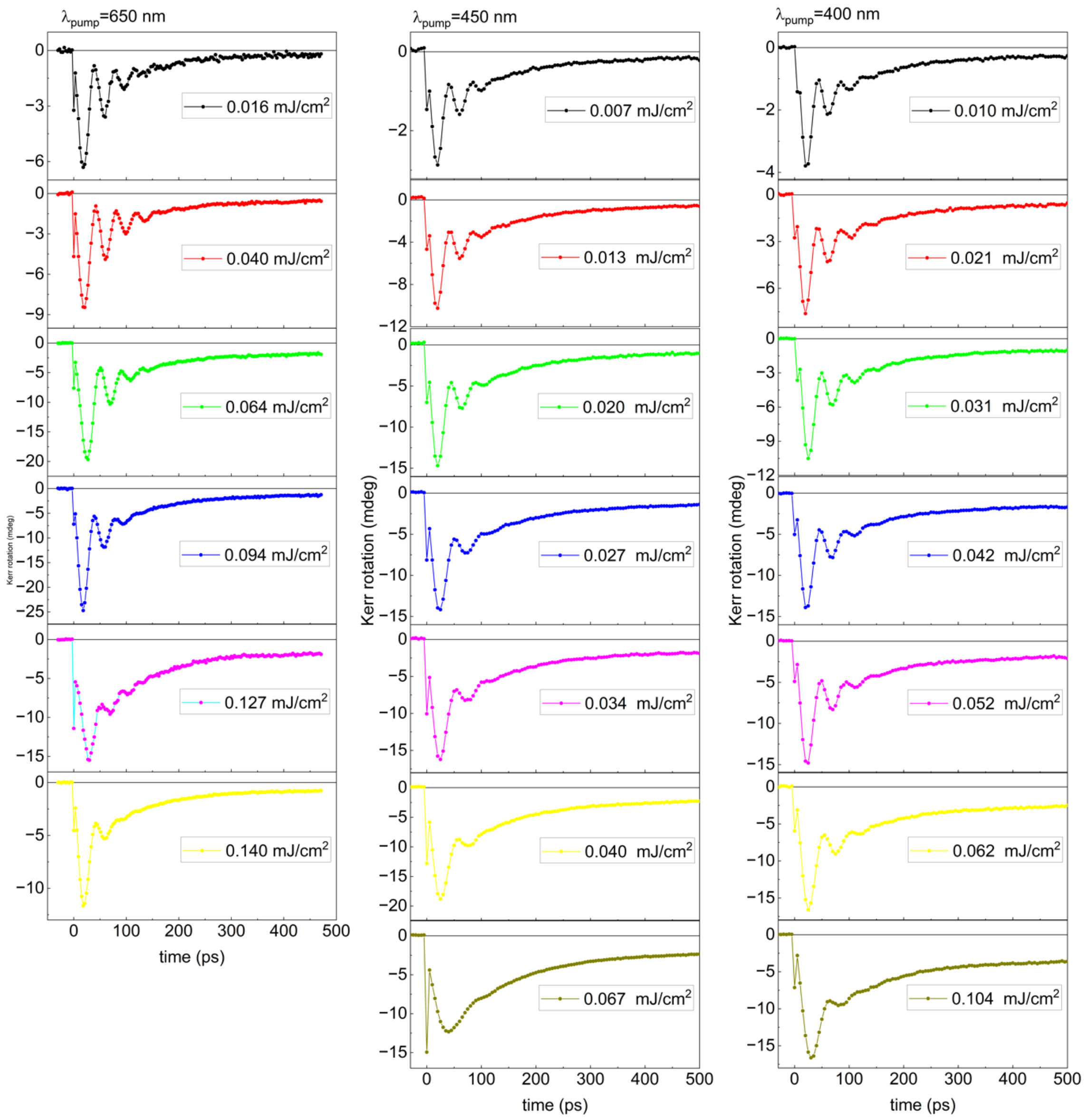

Figure S3 Raw tr-MOKE data obtained from the Co/C_{60} sample at T=80K for pump wavelengths of 650 nm (1.9 eV), 450 nm (2.75 eV), and 400 nm (3.1 eV).

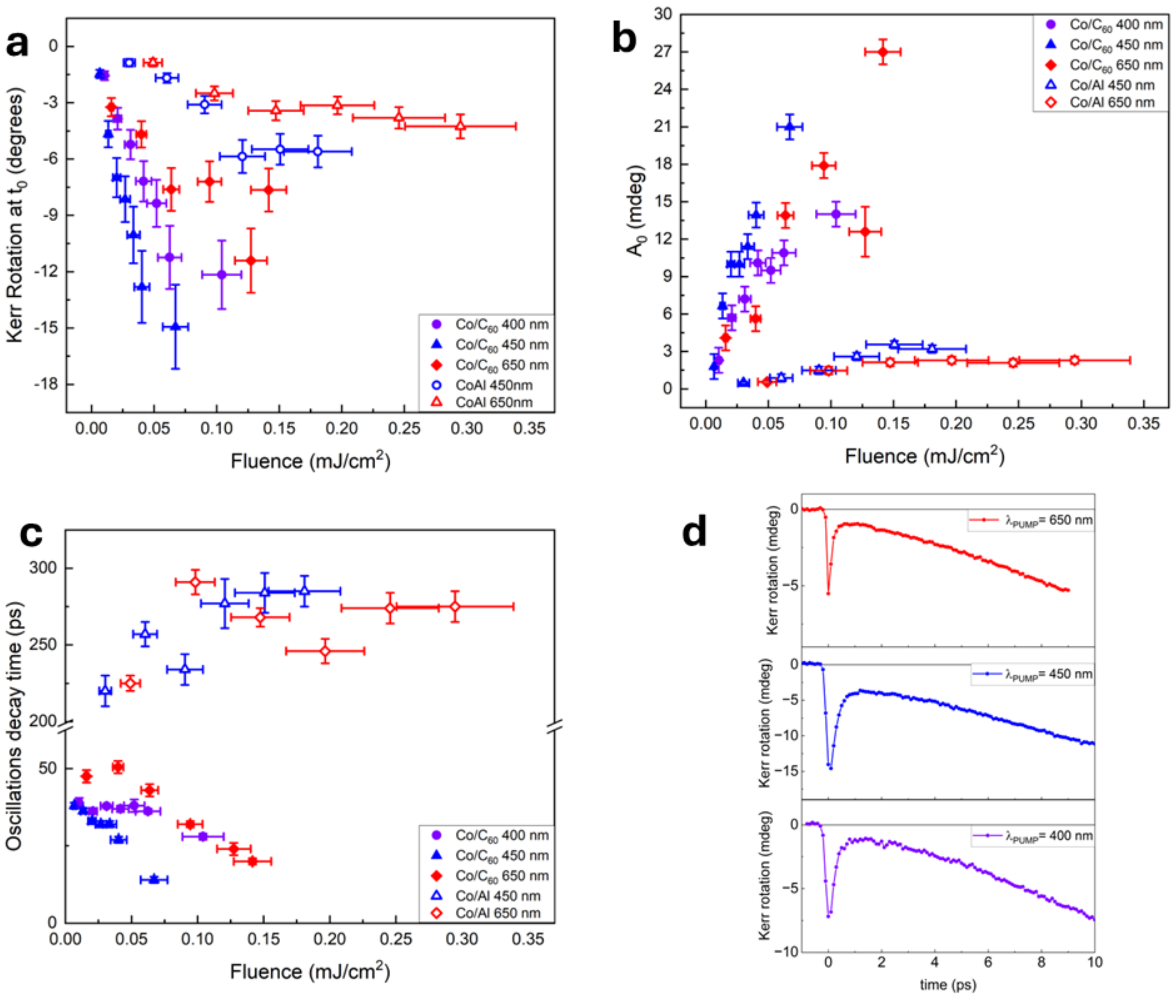

Figure S4 Extracted parameters from tr-MOKE data for Co/Al (hollow points) and Co/C_{60} (solid points) samples. **a**, pump-induced ultrafast magnetization peak. **b**, extracted values of A_0 for each pump wavelength used, as a function of fluence. **c**, Extracted decay time τ_D (**c**) for each pump wavelength, as a function of fluence. **d**, tr-MOKE traces for the Co/C_{60} sample showing the pump-induced ultrafast demagnetization for an exemplarily effective fluence of 0.045 mJ/cm^2.

S.III Time-dependent oscillation frequency and high fluence data

We now discuss the possibility of a time-dependent oscillation frequency when the optical pump is resonantly absorbed by the C_{60} layer. **Fig. S5** presents the tr-MOKE trace for Co/C_{60} excited by 450 nm pump wavelength, for a fluence of 0.033 mJ/cm^2, with the background subtracted. Fitting the data with a single damped sinusoidal function accurately reproduces the first half period but introduces an apparent delay compared to the experimental data at later times. This discrepancy can be interpreted as a gradual recovery of the system's effective anisotropy field, driven by the time-dependent decay of excitons in the C_{60} layer. However, due to the strong damping of the dynamical response, this time dependence is largely obscured and can be considered negligible to a first approximation.

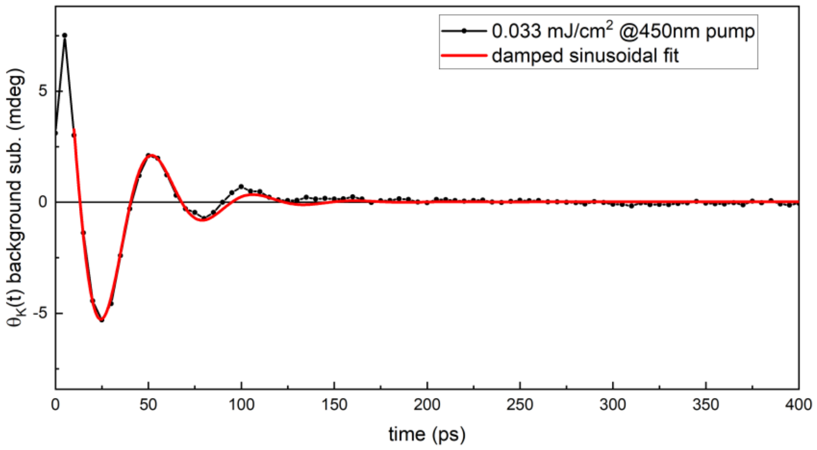

Fig. S5 Background subtracted tr-MOKE trace (black dots) of Co/C_{60} sample, $\lambda_{pump} = 450\,nm, w_{Co} \approx 0.033\,mJ/cm^2$ together with the damped sinusoidal fit function used to extract the oscillation parameters.

In the manuscript, we have concentrated on the data obtained in the low-fluence regime, defined as the region where the oscillation frequency depends linearly on the adsorbed fluence. We now discuss the effects observed at higher fluences. As shown in **Fig. S6**, after ultrafast demagnetization, at higher fluencies the MOKE signal shows additional dynamics at intermediate timescales, happening concurrently with the uniform coherent damped oscillation mode. To analyse these dynamics, we fitted the data using two different models. The first model consists of a single exponential term combined with two damped sinusoidal functions:

$$\theta_K(t) = \theta_0 + A_E \exp\left(-\frac{t}{\tau_E}\right) + A_1 \exp\left(-\frac{t}{\tau_1}\right)\sin\left(2\pi\nu_2(t-t_0)\right)$$
$$+ A_2 \exp\left(-\frac{t}{\tau_2}\right)\sin\left(2\pi\nu_2(t-t_0)\right) \quad (S6)$$

The second model is based on Eq. (S5) but with one exponential having a positive amplitude value at 0. We found that eq. (S6) best reproduces the data. The extracted parameters are reported in **Table S1** and **Table S2**, together with the error values obtained from the fit. The fitting accuracy statistically indicates the presence of a second oscillation mode, with low frequency ($\nu_2 \approx 4\ GHz$) and strong damping, that we do not further discuss here. We point out that, even in this data set, we observe a dependence of the uniform precession mode frequency (ν_1) on the excitation wavelength, being reduced by a factor 4/10 when resonant optical excitation of the C_{60} layer takes place (see **Table S1** and **Table S2**), in line with the low-fluence data. These data can also be used to test the possibility of a time-dependent oscillation frequency.

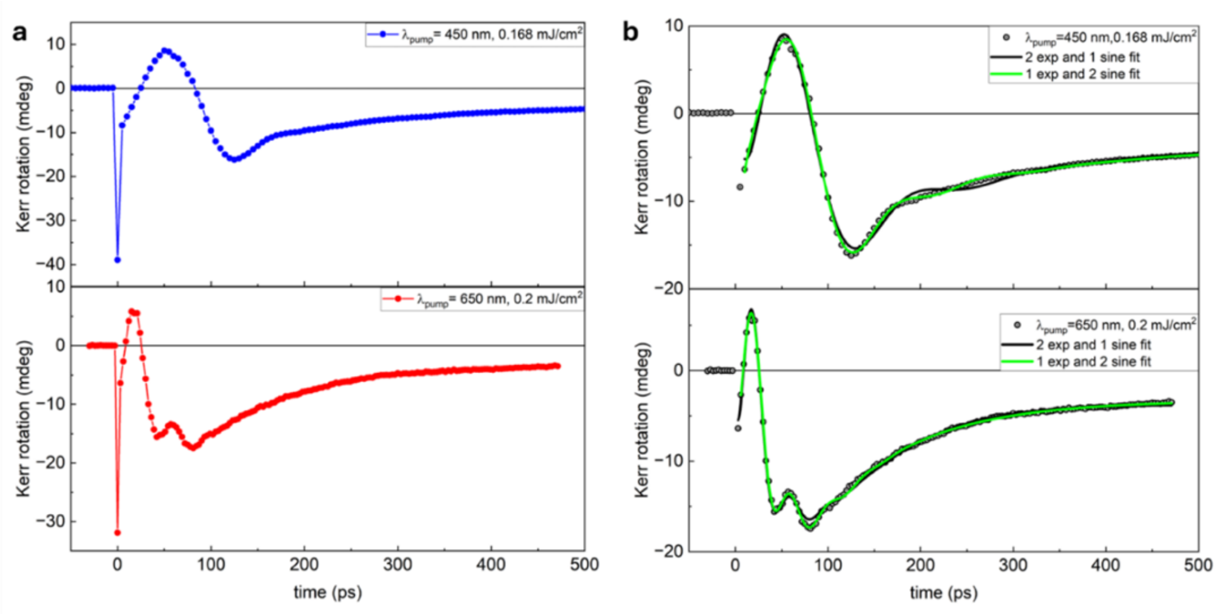

Figure S6 a, high fluence tr-MOKE traces of the Co/C_{60} system after photoexcitation with a 450 nm (top) and 650 nm pump (bottom). The fitting curves can be seen in **b**.

Table S1 *extracted values of the fit parameters, single exponent + 2 sinusoids.*

Parameter	650 nm pump	450 nm pump
A_E (mdeg)	-31 ± 2	-16.2 ± 0.4
$\boldsymbol{\tau_E}$ (ps)	103 ± 4	195 ± 4
A_1 (mdeg)	-14.6 ± 0.6	-21 ± 1
$\boldsymbol{\tau_1}$ (ps)	30 ± 1	59 ± 2
ν_1 (GHz)	24.8 ± 0.06	9.94 ± 0.051
A_2 (mdeg)	57 ± 9	90 ± 5
$\boldsymbol{\tau_2}$ (ps)	28 ± 2	41 ± 1
ν_2 (GHz)	3.8 ± 0.08	4.43 ± 0.04

Table S2 extracted values of the fit parameters, 2 exponents + 1 sinusoidal.

Parameter	650 nm pump	450 nm pump
A_1 (mdeg)	-154 ± 123	-27 ± 3
T_1 (ps)	63 ± 7	146 ± 8
A_2 (mdeg)	174 ± 123	72 ± 3
T_2 (ps)	46 ± 6	41 ± 3
A_0 (mdeg)	21.6 ± 0.6	-42.6
T_{DS} (ps)	22 ± 0.7	41 ± 1
ν_{DS} (GHz)	24.8 ± 0.1	7.42 ± 0.04

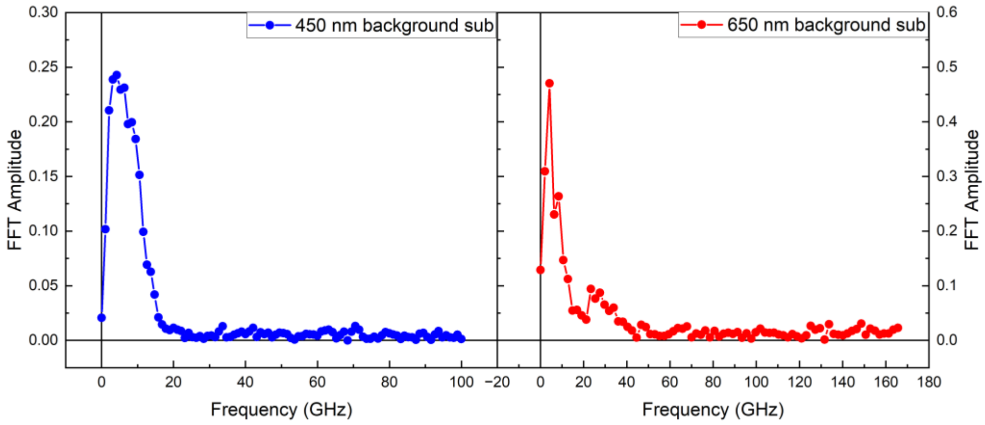

Figure S7: FFT analysis of the high-fluence tr-MOKE traces reported in **Fig. S5**.

In **Fig. S7** we show the FFT of the high fluence data after removal of the long-timescale background exponential term (Hanning window applied). In the off-resonance case (650 nm) we observe a first peak around 5 GHz and a second, weaker, at around 25 GHz. The latter is absent for the *on-resonance* case (450 nm) where we observe a very broad peak centred at around 7 GHz, which is compatible with the presence of a time-dependent oscillation frequency. This confirms a possible coexistence of two modes, a first low-frequency pump-wavelength-independent mode and a second one which is strongly quenched when absorption by C_{60} takes place. A more comprehensive understanding of the rich magnetization dynamics observed at high fluence is beyond the scope of this paper and will be the subject of future investigations.

S.IV Choice of the optimal cobalt and C_{60} layers thickness

A 25 nm thick C_{60} layer thickness was chosen in order to ensure full and homogeneous coverage of the Co layer. Such thickness is not expected to influence the spin precession frequency. Previous studies[7,8] have shown that only the first molecular layer—and to some extent, the second—participates in the formation of a hybrid interface with the transition metal. Specifically, the first C_{60} monolayer undergoes hybridization, leading to a broadening and spin-splitting of its molecular orbitals. The second monolayer can develop interface-induced electronic states with a spin-dependent lifetime. Beyond the second monolayer, additional C_{60} layers do not significantly contribute to the interfacial electronic or magnetic properties.

We experimentally verified the impact of Co thickness and of a Co/C_{60} interface by measuring samples with different Co layer thicknesses before performing the tr-MOKE experiments presented in the main text. These measurements allowed us to determine the optimal Co thickness for maximizing the Kerr response, while allowing for a strong interfacial effect on the Co magnetic anisotropy. This can be seen in **Fig. S8** where we report the in-plane hysteresis loops for Co/C_{60} with co thickness of 3 and 5 nm, along with a reference Co(5nm)/Al(3nm), measured at T=80 K. The effectiveness of the molecular interface on the magnetic hardening can be inferred by comparing the coercive field of the Co(5nm)/C_{60}(25nm) sample, (48 ± 2) mT, with the (4.6 ± 0.4) mT coercivity of the Co(5nm)/Al(3nm) sample. Data for 7-nm-thick Co layers can be found in Ref. [9] . While the hardening effect is still visible, it is greatly reduced in magnitude.

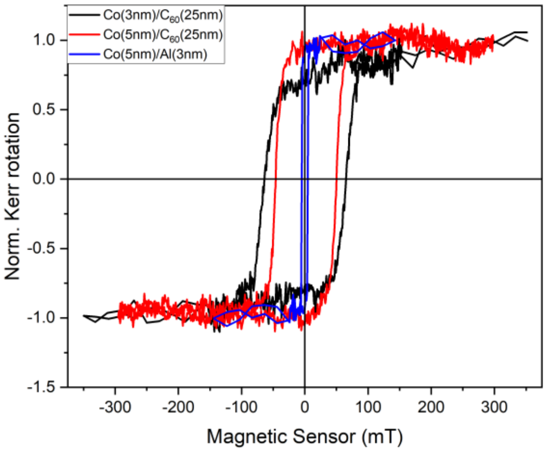

Figure S8 *Hysteresis loops of the Co/C_{60} samples, compared with a reference Co(5nm)/Al(3 nm) sample.*

S.V Temperature dependent absorbance spectra

In order to gain more insight into the temperature dependent characteristics of the excitons, we performed optical absorption spectroscopy at various temperatures. **Fig. S9** shows the spectra between 350 and 800 nm. In **Fig. S10**, we present the zoomed-in spectra from the exciton signal, which was fitted with three Gaussian peaks.

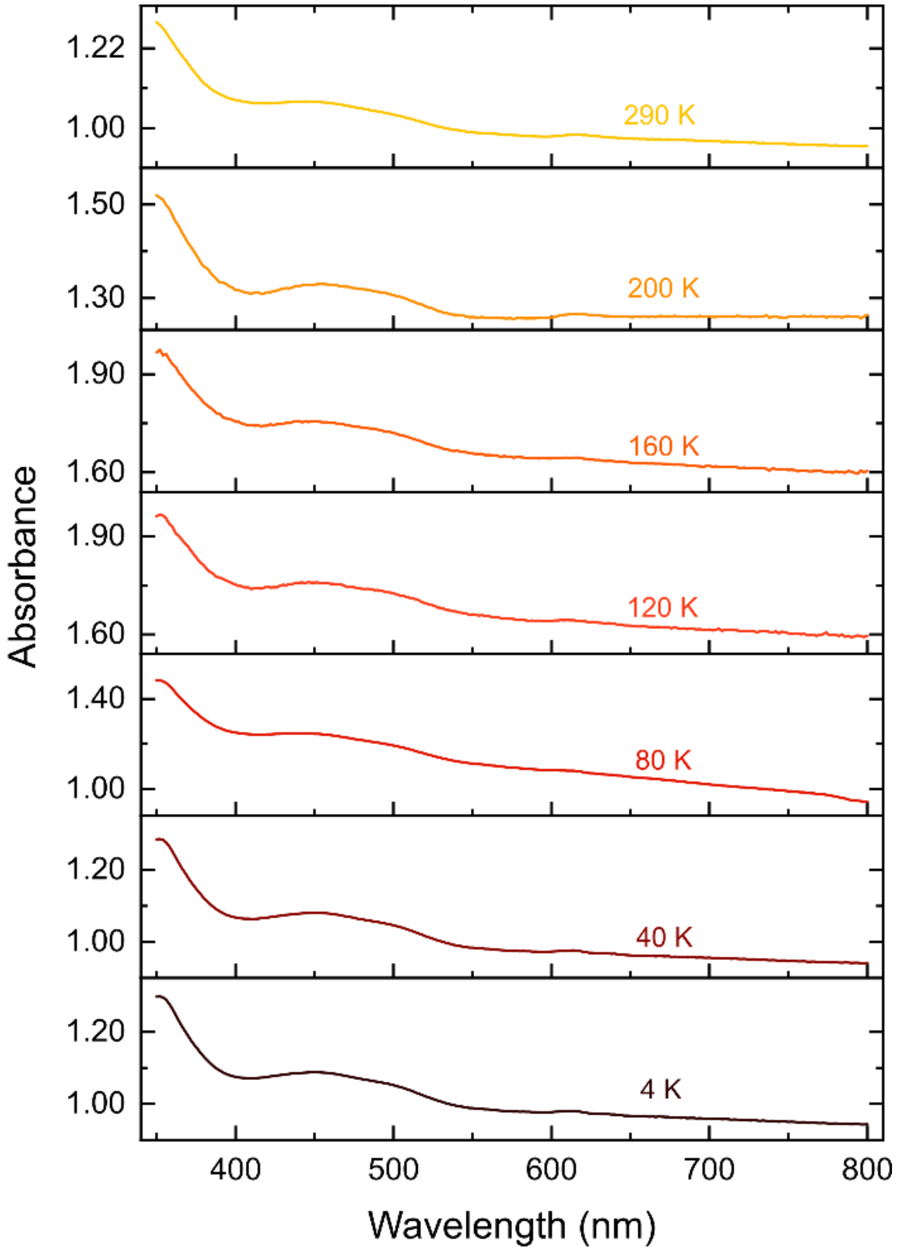

Figure S.9 Temperature-dependent absorbance data obtained from Co/C_{60} sample.

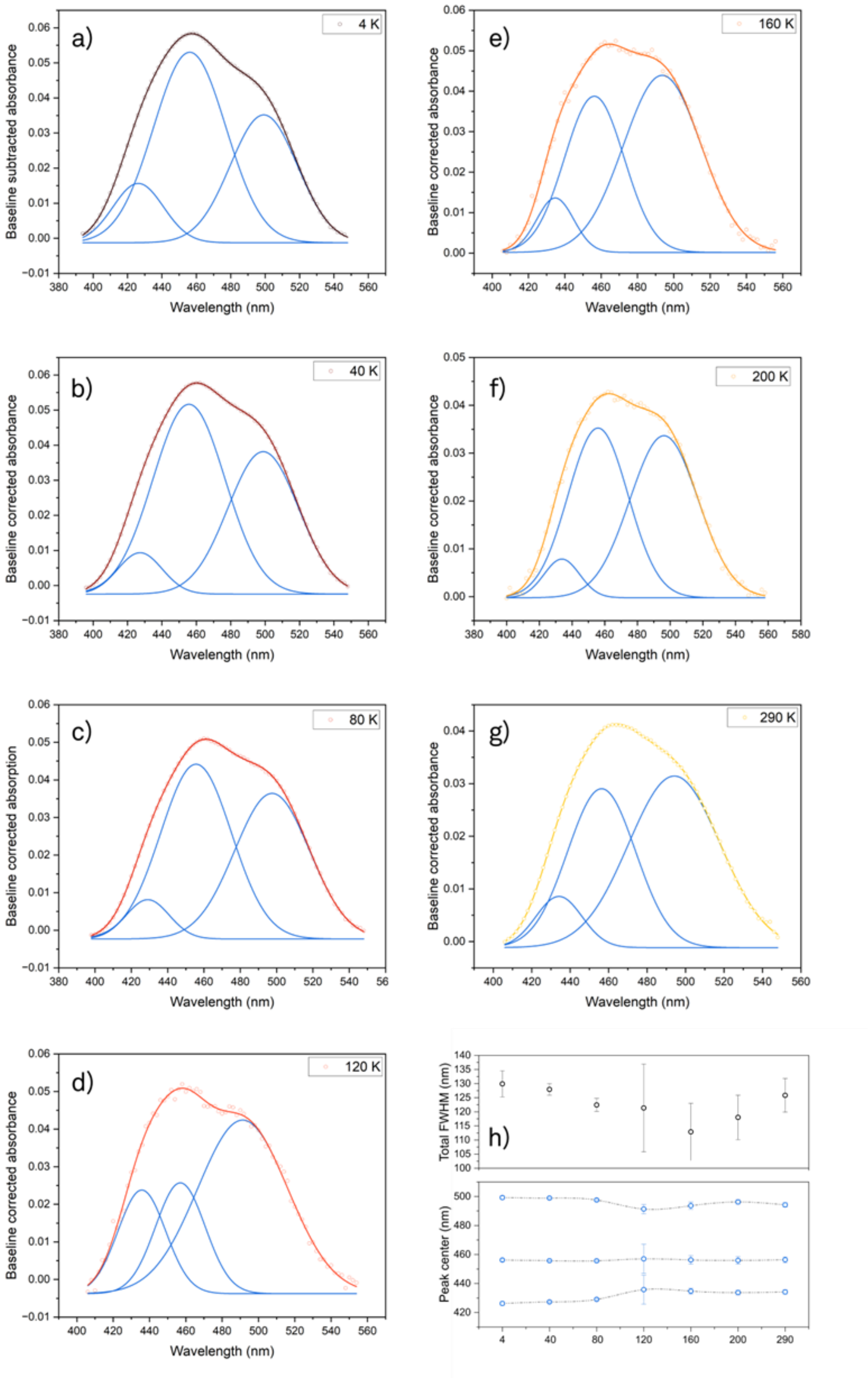

Fig. S10 (a)-(g) Baseline corrected absorbance within the exciton absorption peak. Data were fitted by three Gaussian peaks, which can be ascribed to the contributions from Frenkel excitons and charge transfer states. (h) Position of the peak centres and the total full width at half maximum (FWHM) of these three peaks as a function of temperature. The lines are guides to the eye. Error bars obtained by fitting procedure.

211

S.VI Dynamics of the Frenkel and charge transfer states

We present a detailed analysis of the transient reflectivity spectrum covering the excitonic response. **Fig. S11** shows the dynamic response of the Co/C_{60} sample after excitation by 351nm and the results of the fit with bi-exponential decay functions. For wavelengths higher than 530nm, the signal contains an additional long-living contribution that decays on a longer timescale than the selected temporal range (>>12ps).

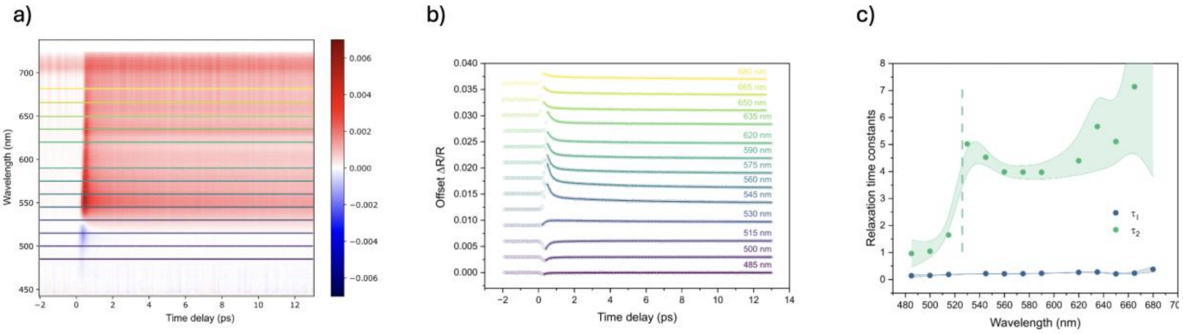

Figure S11 Relaxation dynamics of the excitonic response. (a) Transient reflectivity spectrum excited by the pump beam at 351 nm. Positive and negative transient reflectivity signals are colour coded by red and blue, respectively. The line profiles are extracted at various spectral components to estimate the relaxation time of different excitonic contributions. (b) Fitting of the data using bi-exponential decay functions, with decay constants τ_1 and τ_2. We note that at 530 nm a single exponential function was used. (c) Extracted decay constants. The dashed line depicts the sign change, which discriminates the contributions of Frenkel and charge transfer states.

To estimate the relaxation rate of the charge transfer excitons we use the decay constant τ_1 extracted from the transient reflectivity curve at 500nm. For the relaxation rate of the formed Frenkel excitons, as well as those directly excited by the pump laser, we use $\tau 2$ = 4.5 ps as extracted from the curve at 540nm.

S.VII Co/C_{60} density of states

Fig. 4b in the manuscript shows the DOS projected on Co surface d orbitals and on the C_{60} HOMO, calculated by Density Functional Theory (DFT) (note that the C_{60} HOMO is five-fold degenerate).

We observe that the HOMO-PDOS is characterized by a sharp peak approximately 1.4 eV below the Fermi energy (E_F) and several side features. The most prominent of these side features appears at energies between -2.2 and -3.5 eV for both spin-up and spin-down electrons, while there is also an additional feature around 0.5 eV, which is more marked for spin-up than spin-down electrons. These side features arise from the formation of bonding and antibonding states between the p orbitals of the C atoms in contact with the surface and the Co d orbitals, as discussed in Ref.[10]. They are responsible for the molecule-Co hybridization. In contrast, the sharp peak is contributed by all other C atoms, indicating that C_{60} largely retains its molecular character, allowing it to display excitonic features.

S.VIII Static out-of-plane characterization

Fig. S12 shows the static MOKE measurements performed at 10 K for the Co/C_{60} and Co/Al bilayers. The measurements indicate that for both samples the film normal axis behaves as a hard axis. For the Co/Al bilayer, the shape anisotropy is indeed expected to dominate over other potential anisotropies that might favor an out-of-plane easy axis[11]. For the Co/C_{60} bilayer our observation agrees with previous findings[8,12], that have additionally shown that although the hybridization between Co and the C_{60} molecules strongly modifies the magnetic anisotropy, the out-of-plane direction remains a hard axis. Note that the different values of the Kerr rotation can be ascribed to a different magneto-optic coupling due to the different refraction indexes between Al and C_{60} [13] and small differences of the incidence angle of the probe beam.

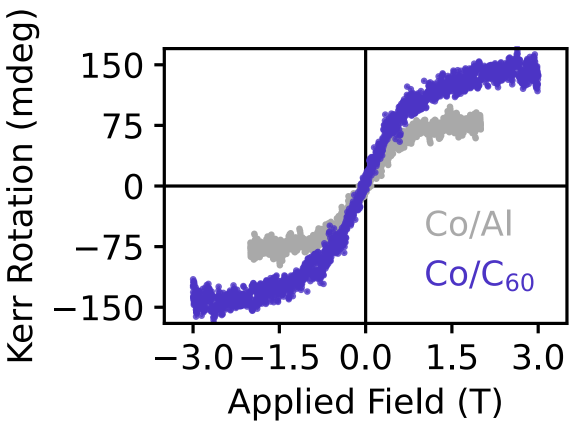

Figure S12 Hysteresis loops recorded at 10 K for Co/Al and Co/C_{60}, demonstrating that the magnetic hard axis matches the normal axis of the film.

References

1. Gan, L. *et al.* Thin Al, Au, Cu, Ni, Fe, and Ta films as oxidation barriers for Co in air. *J. Appl. Phys.* **93**, 8731–8733 (2003).

2. Johnson, B. I. *et al.* Using ellipsometry and x-ray photoelectron spectroscopy for real-time monitoring of the oxidation of aluminum mirrors protected by ultrathin MgF2 layers. *Astron. Opt.: Des., Manuf., Test Space Ground Syst. II* **11116**, 111160O-111160O–13 (2019).

3. Mertens, F. *et al.* Wide spectral range ultrafast pump–probe magneto-optical spectrometer at low temperature, high-magnetic and electric fields. *Review of Scientific Instruments* 1–9 (2020) doi:10.1063/5.0024449.

4. Chudnovsky, E. M. & Garanin, D. A. Static and microwave properties of amorphous magnets near saturation. *Eur. Phys. J. B* **97**, 186 (2024).

5. Benini, M. *et al.* The Ferromagnetic Glass State: collapse of the standard ferromagnetic domain structure. (2024) doi:10.21203/rs.3.rs-4540787/v1.

6. Koopmans, B. *et al.* Explaining the paradoxical diversity of ultrafast laser-induced demagnetization. *Nature Materials* **9**, 259–265 (2009).

7. Droghetti, A. *et al.* Dynamic spin filtering at the Co/Alq3 interface mediated by weakly coupled second layer molecules. *Nat Commun* **7**, 1 9 (2016).

8. Moorsom, T. *et al.* π-anisotropy: A nanocarbon route to hard magnetism. *Phys. Rev. B* **101**, 060408 (2020).

9. Benini, M. *et al.* In-Depth NMR Investigation of the Magnetic Hardening in Co Thin Films Induced by the Interface with Molecular Layers. *Adv. Mater. Interfaces* **9**, (2022).

10. Halder, A., Bhandary, S., O'Regan, D. D., Sanvito, S. & Droghetti, A. Theoretical perspective on the modification of the magnetocrystalline anisotropy at molecule-cobalt interfaces. *Phys. Rev. Mater.* **7**, 064409 (2023).

11. Chappert, C., Dang, K. L., Beauvillain, P., Hurdequint, H. & Renard, D. Ferromagnetic resonance studies of very thin cobalt films on a gold substrate. *Phys. Rev. B* **34**, 3192–3197 (1986).

12. Moorsom, T. *et al.* Spin-polarized electron transfer in ferromagnet/C60interfaces. *Physical Review B* **90**, 125311–6 (2014).

13. Ahn, K. & Fan, G. Kerr effect enhancement in ferromagnetic films. *IEEE Trans. Magn.* **2**, 678–680 (1966).
